# 3-Hydr­oxy-*N*′-isopropyl­idene-2-naphthohydrazide

**DOI:** 10.1107/S1600536809050661

**Published:** 2009-11-28

**Authors:** See Mun Lee, Kong Mun Lo, Hapipah Mohd Ali, Seik Weng Ng

**Affiliations:** a Department of Chemistry, University of Malaya, 50603 Kuala Lumpur, Malaysia

## Abstract

The title Schiff base, C_14_H_14_N_2_O_2_, is close to being planar (r.m.s. deviation for the non-hydrogen atoms = 0.052 Å) and an intra­molecular N—H⋯O hydrogen bond generates an *S*(6) ring. In the crystal, the moleucles are linked by O—H⋯O hydrogen bonds, giving rise to helical chains propagating along the *c* axis of the tetra­gonal unit cell.

## Related literature

For the crystal structure of 2′-(2-isopropyl­idene)-2-hydroxy­benzohydrazide monohydrate, see: Kraudelt *et al.* (1996 ▶).
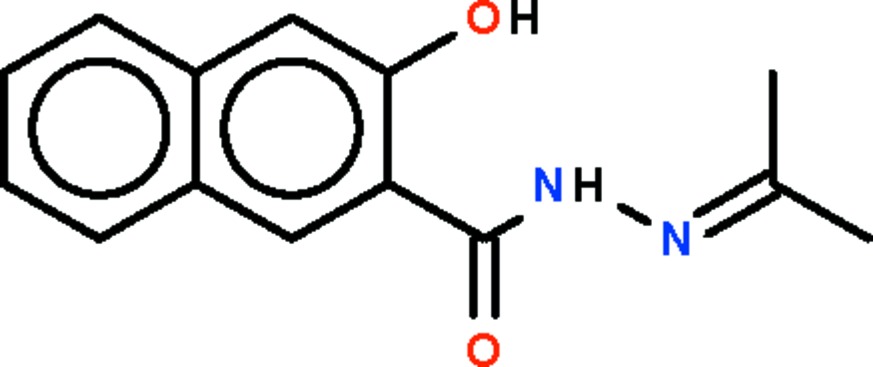


## Experimental

### 

#### Crystal data

C_14_H_14_N_2_O_2_
*M* *_r_* = 242.27
Tetragonal, 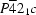

*a* = 13.7343 (3) Å
*c* = 12.8253 (3) Å
*V* = 2419.25 (8) Å^3^
*Z* = 8
Mo *K*α radiation
μ = 0.09 mm^−1^
*T* = 123 K
0.35 × 0.15 × 0.05 mm

#### Data collection

Bruker SMART APEX diffractometer
16460 measured reflections
1566 independent reflections
1341 reflections with *I* > 2σ(*I*)
*R*_int_ = 0.055

#### Refinement

*R*[*F* ^2^ > 2σ(*F* ^2^)] = 0.041
*wR*(*F* ^2^) = 0.113
*S* = 1.01
1566 reflections
173 parameters
2 restraints
H atoms treated by a mixture of independent and constrained refinement
Δρ_max_ = 0.27 e Å^−3^
Δρ_min_ = −0.22 e Å^−3^

### 

Data collection: *APEX2* (Bruker, 2008 ▶); cell refinement: *SAINT* (Bruker, 2008 ▶); data reduction: *SAINT*; program(s) used to solve structure: *SHELXS97* (Sheldrick, 2008 ▶); program(s) used to refine structure: *SHELXL97* (Sheldrick, 2008 ▶); molecular graphics: *X-SEED* (Barbour, 2001 ▶); software used to prepare material for publication: *publCIF* (Westrip, 2009 ▶).

## Supplementary Material

Crystal structure: contains datablocks global, I. DOI: 10.1107/S1600536809050661/hb5249sup1.cif

Structure factors: contains datablocks I. DOI: 10.1107/S1600536809050661/hb5249Isup2.hkl

Additional supplementary materials:  crystallographic information; 3D view; checkCIF report

## Figures and Tables

**Table 1:** Hydrogen-bond geometry (Å, °)

*D*—H⋯*A*	*D*—H	H⋯*A*	*D*⋯*A*	*D*—H⋯*A*
O1—H1o⋯O2^i^	0.84 (1)	1.87 (2)	2.648 (2)	153 (3)
N1—H1n⋯O1	0.88 (1)	1.93 (2)	2.631 (2)	136 (2)

Symmetry code: (i) 
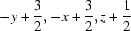
.

**Fig. 1.**
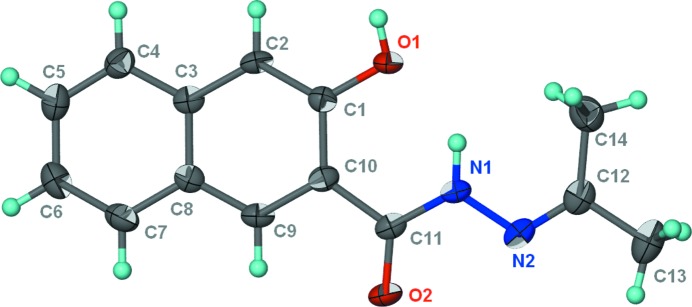
The molecular structure of (I) at the 70% probability level; hydrogen atoms are drawn as spheres of arbitrary radius.

## supplementary crystallographic information

### Experimental

The title Schiff base was obtained as a side product from the reaction between 3-hydroxy-2-naphthoic hydrazide (1 g, 5 mmol) and 4-chlorobenzaldehyde (0.7 g, 5 mmol) in acetone. Colourless irregular chunks of (I) were obtained when the solvent was allowed to evaporate slowly.

### Refinement

Anomalous dispersion was negligible and Friedel pairs were merged before refinement.

Carbon-bound H-atoms were placed in calculated positions (C–H 0.95–0.98 Å) and were included in the refinement in the riding model approximation, with *U*(H) set to 1.2–1.5*U*(C). The amino and hydroxy H-atoms were located in a difference Fourier map and were refined with distance restraints of N–H 0.88±0.01 Å and O–H 0.84±0.01 Å.

### Crystal data

**Table:**

C_14_H_14_N_2_O_2_	*D*_x_ = 1.330 Mg m^−^^3^
*M**_r_* = 242.27	Mo *K*α radiation, λ = 0.71073 Å
Tetragonal, *P*42_1_*c*	Cell parameters from 2887 reflections
Hall symbol: P -42 n	θ = 2.2–26.0°
*a* = 13.7343 (3) Å	µ = 0.09 mm^−^^1^
*c* = 12.8253 (3) Å	*T* = 123 K
*V* = 2419.25 (8) Å^3^	Irregular, colourless
*Z* = 8	0.35 × 0.15 × 0.05 mm
*F*(000) = 1024	

### Data collection

**Table:**

Bruker SMART APEX diffractometer	1341 reflections with *I* > 2σ(*I*)
Radiation source: fine-focus sealed tube	*R*_int_ = 0.055
graphite	θ_max_ = 27.5°, θ_min_ = 2.1°
ω scans	*h* = −17→17
16460 measured reflections	*k* = −17→17
1566 independent reflections	*l* = −16→16

### Refinement

**Table:**

Refinement on *F*^2^	Secondary atom site location: difference Fourier map
Least-squares matrix: full	Hydrogen site location: inferred from neighbouring sites
*R*[*F*^2^ > 2σ(*F*^2^)] = 0.041	H atoms treated by a mixture of independent and constrained refinement
*wR*(*F*^2^) = 0.113	*w* = 1/[σ^2^(*F*_o_^2^) + (0.0711*P*)^2^ + 0.6133*P*] where *P* = (*F*_o_^2^ + 2*F*_c_^2^)/3
*S* = 1.01	(Δ/σ)_max_ = 0.001
1566 reflections	Δρ_max_ = 0.27 e Å^−^^3^
173 parameters	Δρ_min_ = −0.21 e Å^−^^3^
2 restraints	Absolute structure: Friedel pairs were merged
Primary atom site location: structure-invariant direct methods	

### Fractional atomic coordinates and isotropic or equivalent isotropic displacement parameters (Å^2^)

**Table:**

	*x*	*y*	*z*	*U*_iso_*/*U*_eq_	
O1	0.89011 (13)	0.64858 (12)	0.81784 (12)	0.0232 (4)	
H1O	0.879 (3)	0.649 (3)	0.8825 (10)	0.047 (10)*	
O2	0.86201 (14)	0.70322 (12)	0.49816 (12)	0.0268 (4)	
N1	0.87509 (16)	0.58826 (14)	0.62381 (16)	0.0213 (4)	
H1N	0.875 (2)	0.5750 (19)	0.6910 (9)	0.026 (8)*	
N2	0.86924 (15)	0.51398 (15)	0.55082 (15)	0.0244 (5)	
C1	0.88285 (16)	0.74227 (16)	0.78288 (18)	0.0185 (5)	
C2	0.88364 (17)	0.81988 (17)	0.85018 (18)	0.0203 (5)	
H2	0.8892	0.8081	0.9229	0.024*	
C3	0.87638 (16)	0.91683 (17)	0.81435 (18)	0.0197 (5)	
C4	0.87394 (18)	0.99834 (18)	0.88226 (19)	0.0241 (5)	
H4	0.8786	0.9887	0.9554	0.029*	
C5	0.86503 (19)	1.09039 (18)	0.8436 (2)	0.0275 (6)	
H5	0.8640	1.1441	0.8902	0.033*	
C6	0.8573 (2)	1.10692 (18)	0.7353 (2)	0.0297 (6)	
H6	0.8505	1.1714	0.7094	0.036*	
C7	0.85970 (19)	1.03001 (18)	0.6677 (2)	0.0273 (6)	
H7	0.8548	1.0413	0.5949	0.033*	
C8	0.86940 (17)	0.93370 (16)	0.70538 (19)	0.0195 (5)	
C9	0.86959 (17)	0.85281 (18)	0.63782 (19)	0.0215 (5)	
H9	0.8657	0.8640	0.5648	0.026*	
C10	0.87513 (17)	0.75829 (17)	0.67298 (18)	0.0192 (5)	
C11	0.87014 (17)	0.68105 (17)	0.59108 (18)	0.0196 (5)	
C12	0.87295 (18)	0.42740 (19)	0.5869 (2)	0.0257 (5)	
C13	0.8674 (2)	0.3471 (2)	0.5088 (2)	0.0374 (7)	
H13A	0.8575	0.3747	0.4392	0.056*	
H13B	0.8129	0.3040	0.5261	0.056*	
H13C	0.9282	0.3098	0.5099	0.056*	
C14	0.8808 (2)	0.39944 (19)	0.7001 (2)	0.0322 (6)	
H14A	0.9250	0.4445	0.7357	0.048*	
H14B	0.9063	0.3330	0.7056	0.048*	
H14C	0.8163	0.4026	0.7325	0.048*	

### Atomic displacement parameters (Å^2^)

**Table:**

	*U*^11^	*U*^22^	*U*^33^	*U*^12^	*U*^13^	*U*^23^
O1	0.0343 (9)	0.0223 (8)	0.0130 (8)	0.0004 (7)	0.0001 (7)	0.0022 (7)
O2	0.0375 (10)	0.0294 (9)	0.0134 (8)	−0.0015 (8)	−0.0012 (8)	−0.0008 (7)
N1	0.0261 (11)	0.0248 (10)	0.0130 (9)	0.0002 (8)	−0.0002 (8)	−0.0008 (8)
N2	0.0253 (10)	0.0272 (10)	0.0205 (10)	−0.0010 (9)	0.0007 (9)	−0.0064 (9)
C1	0.0166 (11)	0.0208 (11)	0.0179 (11)	−0.0003 (9)	0.0005 (9)	0.0015 (9)
C2	0.0195 (12)	0.0284 (12)	0.0130 (10)	−0.0013 (10)	−0.0018 (9)	−0.0009 (9)
C3	0.0139 (10)	0.0254 (11)	0.0198 (11)	−0.0003 (9)	−0.0002 (9)	0.0000 (10)
C4	0.0235 (12)	0.0253 (12)	0.0235 (12)	0.0007 (10)	−0.0026 (10)	−0.0041 (10)
C5	0.0265 (13)	0.0223 (12)	0.0337 (14)	0.0017 (11)	−0.0006 (12)	−0.0053 (11)
C6	0.0296 (13)	0.0227 (12)	0.0368 (14)	0.0015 (10)	0.0010 (12)	0.0052 (11)
C7	0.0313 (14)	0.0279 (13)	0.0226 (12)	0.0020 (11)	−0.0009 (11)	0.0070 (10)
C8	0.0147 (10)	0.0228 (11)	0.0211 (11)	−0.0004 (9)	−0.0016 (9)	0.0014 (9)
C9	0.0201 (11)	0.0288 (12)	0.0155 (10)	−0.0003 (10)	−0.0003 (9)	0.0027 (10)
C10	0.0167 (11)	0.0242 (11)	0.0168 (11)	−0.0018 (9)	−0.0005 (9)	−0.0014 (9)
C11	0.0160 (10)	0.0262 (12)	0.0167 (11)	−0.0017 (9)	0.0004 (9)	−0.0025 (9)
C12	0.0198 (11)	0.0274 (12)	0.0298 (13)	−0.0026 (10)	−0.0002 (11)	−0.0052 (10)
C13	0.0339 (14)	0.0336 (14)	0.0446 (16)	0.0000 (12)	0.0029 (14)	−0.0146 (13)
C14	0.0337 (14)	0.0281 (13)	0.0348 (15)	−0.0026 (11)	−0.0025 (12)	0.0035 (12)

### Geometric parameters (Å, °)

**Table:**

O1—C1	1.366 (3)	C6—C7	1.367 (4)
O1—H1O	0.843 (10)	C6—H6	0.9500
O2—C11	1.235 (3)	C7—C8	1.414 (3)
N1—C11	1.344 (3)	C7—H7	0.9500
N1—N2	1.387 (3)	C8—C9	1.409 (3)
N1—H1N	0.880 (10)	C9—C10	1.376 (3)
N2—C12	1.277 (3)	C9—H9	0.9500
C1—C2	1.372 (3)	C10—C11	1.494 (3)
C1—C10	1.430 (3)	C12—C13	1.492 (4)
C2—C3	1.412 (3)	C12—C14	1.505 (4)
C2—H2	0.9500	C13—H13A	0.9800
C3—C8	1.420 (3)	C13—H13B	0.9800
C3—C4	1.419 (3)	C13—H13C	0.9800
C4—C5	1.364 (4)	C14—H14A	0.9800
C4—H4	0.9500	C14—H14B	0.9800
C5—C6	1.411 (4)	C14—H14C	0.9800
C5—H5	0.9500		
			
C1—O1—H1O	108 (2)	C9—C8—C3	118.5 (2)
C11—N1—N2	118.94 (19)	C7—C8—C3	119.7 (2)
C11—N1—H1N	120.1 (18)	C10—C9—C8	122.8 (2)
N2—N1—H1N	120.6 (18)	C10—C9—H9	118.6
C12—N2—N1	116.0 (2)	C8—C9—H9	118.6
O1—C1—C2	121.7 (2)	C9—C10—C1	118.2 (2)
O1—C1—C10	118.27 (19)	C9—C10—C11	115.9 (2)
C2—C1—C10	120.1 (2)	C1—C10—C11	125.9 (2)
C1—C2—C3	121.8 (2)	O2—C11—N1	122.7 (2)
C1—C2—H2	119.1	O2—C11—C10	120.5 (2)
C3—C2—H2	119.1	N1—C11—C10	116.8 (2)
C2—C3—C8	118.6 (2)	N2—C12—C13	116.3 (2)
C2—C3—C4	123.1 (2)	N2—C12—C14	126.2 (2)
C8—C3—C4	118.3 (2)	C13—C12—C14	117.5 (2)
C5—C4—C3	120.7 (2)	C12—C13—H13A	109.5
C5—C4—H4	119.7	C12—C13—H13B	109.5
C3—C4—H4	119.7	H13A—C13—H13B	109.5
C4—C5—C6	120.9 (2)	C12—C13—H13C	109.5
C4—C5—H5	119.5	H13A—C13—H13C	109.5
C6—C5—H5	119.5	H13B—C13—H13C	109.5
C7—C6—C5	119.9 (2)	C12—C14—H14A	109.5
C7—C6—H6	120.1	C12—C14—H14B	109.5
C5—C6—H6	120.1	H14A—C14—H14B	109.5
C6—C7—C8	120.6 (2)	C12—C14—H14C	109.5
C6—C7—H7	119.7	H14A—C14—H14C	109.5
C8—C7—H7	119.7	H14B—C14—H14C	109.5
C9—C8—C7	121.9 (2)		
			
C11—N1—N2—C12	−179.0 (2)	C7—C8—C9—C10	−177.4 (2)
O1—C1—C2—C3	179.9 (2)	C3—C8—C9—C10	0.8 (4)
C10—C1—C2—C3	0.4 (4)	C8—C9—C10—C1	−1.2 (4)
C1—C2—C3—C8	−0.9 (4)	C8—C9—C10—C11	177.5 (2)
C1—C2—C3—C4	178.0 (2)	O1—C1—C10—C9	−178.9 (2)
C2—C3—C4—C5	−178.8 (2)	C2—C1—C10—C9	0.6 (4)
C8—C3—C4—C5	0.1 (4)	O1—C1—C10—C11	2.6 (4)
C3—C4—C5—C6	0.5 (4)	C2—C1—C10—C11	−177.9 (2)
C4—C5—C6—C7	−0.6 (4)	N2—N1—C11—O2	−1.1 (4)
C5—C6—C7—C8	0.3 (4)	N2—N1—C11—C10	179.0 (2)
C6—C7—C8—C9	178.4 (2)	C9—C10—C11—O2	0.3 (3)
C6—C7—C8—C3	0.2 (4)	C1—C10—C11—O2	178.9 (2)
C2—C3—C8—C9	0.3 (4)	C9—C10—C11—N1	−179.8 (2)
C4—C3—C8—C9	−178.6 (2)	C1—C10—C11—N1	−1.2 (4)
C2—C3—C8—C7	178.5 (2)	N1—N2—C12—C13	−179.6 (2)
C4—C3—C8—C7	−0.4 (4)	N1—N2—C12—C14	1.4 (4)

### Hydrogen-bond geometry (Å, °)

**Table:**

*D*—H···*A*	*D*—H	H···*A*	*D*···*A*	*D*—H···*A*
O1—H1o···O2^i^	0.84 (1)	1.870 (19)	2.648 (2)	153 (3)
N1—H1n···O1	0.88 (1)	1.93 (2)	2.631 (2)	136 (2)

Symmetry codes: (i) −*y*+3/2, −*x*+3/2, *z*+1/2.
